# Discovering misannotated lncRNAs using deep learning training dynamics

**DOI:** 10.1093/bioinformatics/btac821

**Published:** 2022-12-26

**Authors:** Afshan Nabi, Berke Dilekoglu, Ogun Adebali, Oznur Tastan

**Affiliations:** Faculty of Engineering and Natural Sciences, Sabanci University, Istanbul 34956, Turkey; Faculty of Engineering and Natural Sciences, Sabanci University, Istanbul 34956, Turkey; Faculty of Engineering and Natural Sciences, Sabanci University, Istanbul 34956, Turkey; Faculty of Engineering and Natural Sciences, Sabanci University, Istanbul 34956, Turkey

## Abstract

**Motivation:**

Recent experimental evidence has shown that some long non-coding RNAs (lncRNAs) contain small open reading frames (sORFs) that are translated into functional micropeptides, suggesting that these lncRNAs are misannotated as non-coding. Current methods to detect misannotated lncRNAs rely on ribosome-profiling (Ribo-Seq) and mass-spectrometry experiments, which are cell-type dependent and expensive.

**Results:**

Here, we propose a computational method to identify possible misannotated lncRNAs from sequence information alone. Our approach first builds deep learning models to discriminate coding and non-coding transcripts and leverages these models’ training dynamics to identify misannotated lncRNAs—i.e. lncRNAs with coding potential. The set of misannotated lncRNAs we identified significantly overlap with experimentally validated ones and closely resemble coding protein sequences as evidenced by significant BLAST hits. Our analysis on a subset of misannotated lncRNA candidates also shows that some ORFs they contain yield high confidence folded structures as predicted by AlphaFold2. This methodology offers promising potential for assisting experimental efforts in characterizing the hidden proteome encoded by misannotated lncRNAs and for curating better datasets for building coding potential predictors.

**Availability and implementation:**

Source code is available at https://github.com/nabiafshan/DetectingMisannotatedLncRNAs.

**Supplementary information:**

Supplementary data are available at *Bioinformatics* online.

## 1 Introduction

Long non-coding RNAs (lncRNAs) are defined as non-coding RNAs greater than 200 nucleotides in length. Functions of most lncRNAs remain unknown; the small fraction that are functionally characterized are known to play vital roles in translation regulation, transcription, chromatin modification and mRNA stability (Batista and Chang, 2013; Rinn and Chang, 2012; Ulitsky and Bartel, 2013). Although lncRNAs—by definition—do not code for proteins, it has been reported that small open reading frames (ORFs) within some lncRNAs are translated into micropeptides of a median length of 23 amino acids (Anfossi and Calin, 2020; Choi *et al.*, 2019; Couso and Patraquim, 2017; Hartford and Lal, 2020; Ingolia *et al.*, 2009; Ji *et al.*, 2015; Lu *et al.*, 2019; Ruiz-Orera *et al.*, 2014; Wang *et al.*, 2020) and perform vital functions across species, including bacteria, flies and humans (Anderson *et al.*, 2015; Hartford and Lal, 2020; Makarewich *et al.*, 2018; Matsumoto *et al.*, 2017; Nelson *et al.*, 2016). The translation events of lncRNAs were overlooked previously because ORFs present in lncRNAs do not meet the conventional criterion of a canonical ORF: that it encodes at least 100 amino acids in eukaryotes (Hartford and Lal, 2020). Identifying misannotated lncRNAs is important for the functional characterization of these transcripts. Moreover, such efforts can lead to more complete census of the proteome.

Mass-spectrometry (MS) is one of the experimental techniques that can be used to detect proteins translated from lncRNAs (Slavoff *et al.*, 2013). However, it has been reported that MS is not as sensitive as transcriptome-based approaches (Chekulaeva and Rajewsky, 2019). One transcriptomics-based approach that can be used to identify putative translated RNAs is ribosome profiling. RNAs undergoing translation are associated with ribosomes; ribosome profiling takes advantage of this fundamental observation and involves capturing and sequencing RNA fragments protected by ribosomes (Ingolia, 2014). Data generated using ribosome profiling has revealed putative coding sORFs within lncRNAs (Ingolia *et al.*, 2009).

Since Ribo-Seq data are known to contain false positives (Ingolia *et al.*, 2009, 2011), several computational methods have been proposed to distinguish true positives from false positives. These include FLOSS (Ingolia, 2014), ORFscore (Bazzini *et al.*, 2014) and PhyloP (Miller *et al.*, 2007; Olexiouk *et al.*, 2018). FLOSS relies on the typical length of Ribo-Seq fragments to determine truly coding Ribo-Seq fragments. ORFscore relies on the property that translating ribosomes shift by three nucleotides (ribosome phasing), which leads to a characteristic pattern wherein true positive fragments have higher sequencing reads every third nucleotide. PhyloP is used to find truly translated Ribo-Seq fragments by probing conservation across species (Miller *et al.*, 2007; Olexiouk *et al.*, 2018). These computational methods applied over ribosome profiling data can be used to find sORFs that are both translated and located within lncRNAs. However, one major limitation of relying on experimental methods to identify misannotated lncRNAs is that not all transcripts are likely to be transcribed and translated at a given time point in a given cell. To obtain a complete picture of the misannotated lncRNAs in the genome, different cell types, at different developmental stages, under different environmental conditions need to be sequenced and analyzed. In contrast, the nucleotide sequence of an lncRNA transcript remains constant across cell types and conditions. Therefore, methods with the ability to detect misannotated lncRNAs from nucleotide sequences alone will be useful, at the very least, in reducing the search space for lncRNA encoded peptides that can then be validated by experimental efforts.

Models to assess the coding potential of an ORF are also available. For instance, logistic regression (Zhu and Gribskov, 2019) and support vector machine (Tong *et al.*, 2020) models have been developed to predict the coding potential of a given sORF with sequence length ≤303 nucleotides. Since data on which sORFs within lncRNAs are coding is sparse, it is impossible to evaluate the performance of these models (Zhu and Gribskov, 2019). This problem is especially relevant for species that are not as well studied. Several classical machine learning (Kang *et al.*, 2017; Kong *et al.*, 2007; Tong and Liu, 2019; Wang *et al.*, 2013) and deep learning (Baek *et al.*, 2018; Camargo *et al.*, 2020; Hill *et al.*, 2018) models, which focus on longer length nucleotide sequences as input, have also been developed to predict the coding potential of a given RNA. For instance, CNIT (Guo *et al.*, 2019), CPC2 (Kang *et al.*, 2017) and CPAT (Wang *et al.*, 2013) generate features based on sequence composition and then train classifiers (XGboost, support vector machine and logistic regression models, respectively) on these features to classify coding versus non-coding sequences. RNASamba (Camargo *et al.*, 2020) uses recurrent neural networks to automatically generate features from sequences and train a classifier to distinguish coding from non-coding RNAs. Most of these methods demonstrate very high prediction performance. However, none of these models addresses the problem that some lncRNAs within the training datasets might be misannotated.

To assist experimental efforts in uncovering the hidden proteome, we present a framework that leverages deep learning models’ training dynamics to determine whether a given lncRNA transcript might have a coding subsequence. This method does not rely on a pre-labeled dataset comprising true positive examples of misannotated lncRNAs, and hence can be applied to any species for which sufficient coding and non-coding examples are available. In particular, we train convolutional neural network (CNN) (LeCun *et al.*, 1989), long short term memory (LSTM) (Hochreiter and Schmidhuber, 1997) and Transformer (Vaswani *et al.*, 2017) architectures to predict whether a given nucleotide sequence is non-coding or coding and adapt the data mapping approach presented by Swayamdipta *et al.* (2020) to identify possible misannotated lncRNAs. Our models can distinguish between coding and non-coding RNAs with average AUC scores up to 94% and AUPR up to 96%. The list of misannotated lncRNAs obtained from these models shows significant overlap with a set of experimentally validated misannotated lncRNAs. Moreover, aggregation of evidence from other resources shows that some of these candidate misannotated lncRNAs have high homology with known proteins, contain known protein domains, and are predicted to have well folded structures. Embedding of coding and non-coding RNAs into lower dimensional space shows that there might be a continuity in the embedded space between coding and misannotated lncRNAs. This approach can be applied to better curate datasets for training coding potential prediction models and can be used alongside Ribo-Seq data to identify misannotated lncRNAs with high confidence.

## 2 Approach

**Fig. 1.**
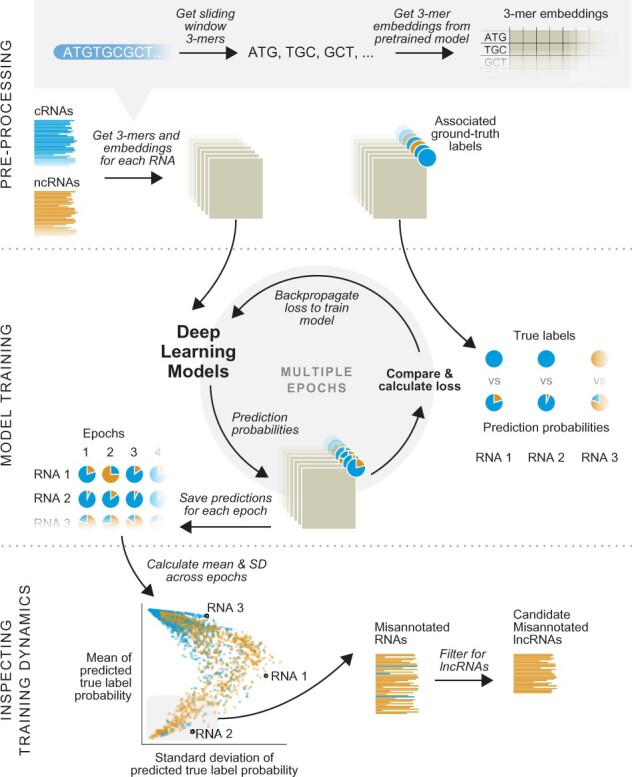
Workflow for identifying misannotated lncRNAs. Upper panel: Each RNA nucleotide sequence is represented as a matrix, where each row is a 3-mer’s 100-dimensional vector embedding. Each 3-mer is obtained by using a sliding window on the RNA sequence and embedding vectors for 3-mers are obtained from the dna2vec model (Ng, 2017). Middle panel: Each RNA also has an associated ground-truth label, coding or non-coding. Deep learning models are trained to predict the coding/non-coding probability for each RNA. Models are detailed in Figure 2. At the end of each training epoch, the predicted probabilities for each RNA are saved. Lower panel: After training, the mean and standard deviation for the ground-truth label probability predictions are calculated. The misannotated lncRNAs are identified based on these mean and standard deviation values

Figure 1 describes the overall workflow we use to determine possibly misannotated lncRNAs. The first step involves encoding RNA nucleotide sequences using pre-trained vector representation. The second step involves training deep learning-based sequence classification models that can distinguish between coding and non-coding RNAs. Once we establish that models can achieve good performance on the held-out test data, we retrain the final models on all the data. In the third step, we inspect the training dynamics of individual RNAs to find misannotated ncRNAs. We detail these steps in the following sections.

## 3 Materials and methods

### 3.1 Datasets

We use the dataset of human RNA nucleotide sequences compiled by Tong and Liu (2019) to train the sequence classification models. After filtering non-coding RNA sequences <200 nucleotides in length, the data comprise 38 051 coding and 19 472 non-coding RNA sequences. Filtering non-coding RNAs by length was necessary since non-coding RNAs are noticeably shorter than coding RNAs. The different length distributions, if not resolved, can become a proxy for distinguishing between coding and non-coding RNAs. We settled on 200 nucleotides as the lower bound for length because lncRNAs are defined as ncRNAs with length >200 nucleotides.

### 3.2 Deep learning model architectures

**Fig. 2.**
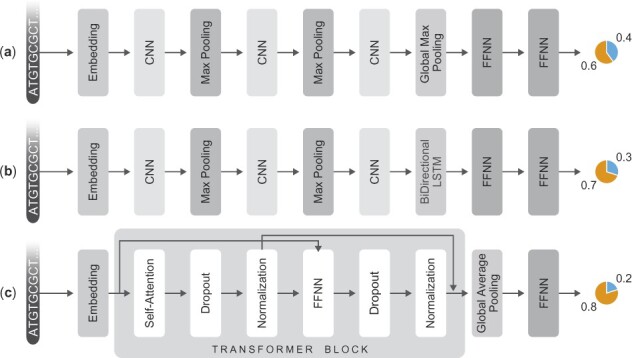
Model architectures. Each model predicts the coding/non-coding probability given an RNA sequence. (**a**) The CNN-based model. (**b**) The LSTM-based model. (**c**) The transformer-based model

We train three different deep learning models: 1D CNN (LeCun *et al.*, 1989), LSTM (Hochreiter and Schmidhuber, 1997) and Transformer (Vaswani *et al.*, 2017) models to classify non-coding and coding RNA sequences. Each input sequence is truncated to a length of 4000 nucleotides before being input to the deep learning models. For each sequence, we obtain sliding window 3-mers. For each 3-mer, we use a 100-dimensional embedding vector obtained from the pretrained model presented in Ng (2017). Thus, each sequence is converted into a matrix of numbers that can be input into the deep learning models, see Figure 1Pre-Processing for more details. We use ReLu as the activation function. We train all models to minimize the sparse categorical cross-entropy loss using the Adam optimizer (Kingma and Ba, 2014). Since the training dataset is imbalanced in favor of coding RNA, we use class-weights inversely proportional to the number of class samples. Moreover, since a coding RNA is unlikely to be misannotated, we penalize coding RNA misclassifications five times more than non-coding RNA misclassifications. In all cases, we use a batch size of 64. All three models are implemented using Keras (https://keras.io/). Figure 2 shows the model architectures used in this work. Architecture specific details are as follows:

**CNN:** For the CNN, encoded sequences are fed into an embedding layer which is followed by 3 layers of 1D convolution, each with 128 units and filter size 5, and max-pooling with 5 units. These are followed by a dense layer of 128 units.

**LSTM:** For the LSTM, encoded sequences are fed into an embedding layer which is followed by 2 layers of 1D convolution (each with 128 units and filter size 5) and max-pooling (5 units), followed by a bidirectional LSTM layer. These are followed by a dense layer of 128 units.

**Transformer:** Encoded sequences are added to a positional encoding and fed into a single transformer block followed by global average pooling, dropout and a dense layer of 64 units. The transformer block contains a single-headed self-attention layer and a dense layer, followed by layer normalization.

### 3.3 Hyperparameter tuning and model evaluation set up

We use the human coding and non-coding train and test datasets provided by Tong and Liu (2019) (see Section 3.1). We set aside 20% of the data as the test data. We use Keras Tuner (https://github.com/keras-team/keras-tuner) on the training set to find the optimal set of hyperparameters for the deep learning models. We created a hyperparameter search space for different model architecture and hyperparameter assignment values and used the Hyperband tuner (Li *et al.*, 2017) to find the optimal parameters based on validation loss. We use the following choices for given hyperparameters: dense layer units 64, 128 and 256; 1D convolutional filters: 64 and 128; LSTM units 64, 128 and 256; dropout: 0.2, 0.3, 0.4 and 0.5; and learning rate: logarithmic sampling between e–2 and e–4. We use the best model returned by the Hyperband tuner and retrain a model on the train-validation data to calculate and assess these models’ performances on the held-out test data. Once the test performances are attained, we rebuilt the models on all data to find the misannotated lncRNAs.

### 3.4 Identifying misannotated lncRNAs using training dynamics

We inspect the deep learning models’ training dynamics to find possible misannotated lncRNAs. We employ the method used in Swayamdipta *et al.* (2020) which relies on inspection of model predictions for each sample across all training epochs. At the end of each training epoch, the deep learning models are evaluated on the training examples and predictions for the class probabilities are saved. Consider a training dataset of size $N,D={\left\{{\left(x,y^{*}\right)}_{i}\right\}}_{i=1}^{N}$, where the *i*th instance consists of the observation, $x_{i}$ and its ground-truth label under the task, $y_{i}^{*}$. We calculate the mean and the standard deviation of the posterior probability of the ground-truth label, for example, *i* over *E* epochs as follows (Swayamdipta *et al.*, 2020):

(1) $$\begin{array}{c} {\hat{\mu }}_{i}=\frac{1}{E}\sum_{e=1}^{E}p_{\theta^{\left(e\right)}}\left(y_{i}^{*}|x_{i}\right)\\ {\hat{\sigma }}_{i}=\sqrt{\frac{\sum_{e=1}^{E}{\left(p_{\theta^{\left(e\right)}}\left(y_{i}^{*}|x_{i}\right)-{\hat{\mu }}_{i}\right)}^{2}}{E}} \end{array},$$

where $p_{\theta^{\left(e\right)}}$ denotes the probability assigned at the end of the *e*th epoch by the model parameterized with $\theta^{\left(e\right)}$. Using the mean and the standard deviation of the predicted probability of ground-truth class across all epochs, the training dataset can be divided into three groups: easy-to-learn, ambiguous and hard-to-learn. The easy-to-learn samples are those for which the model predicts with high confidence that the labels are correct, as evidenced by the high mean and low standard deviation in the predictions for the ground truth class. In contrast, hard-to-learn samples are those with low mean and low standard deviation of the ground truth class. In other words, the model consistently misclassifies these samples across training epochs. We retrain the models using both the training and test data and consider hard-to-learn lncRNAs as candidates for misannotation.

In deciding which examples could be misannoated, one important question is how the predictions of different epochs should be weighed when identifying mislabeled samples. Equation (1) considers all epochs with equal weight. However, is it better to value later epoch predictions more highly than earlier epoch predictions? In order to answer this question, different schemes were used when calculating the mean and standard deviation of epoch predictions: (i) weigh all epochs equally (referred to as base) as in Equation (1); (ii) weigh epochs in proportion to the epoch number such that earlier epochs get lower weight (referred to as weighted); and (iii) ignore first 5%, 10%, 20%, 40%, 60% or 80% epochs (referred to as ignore_1st_x where x is the percent of initial epochs ignored). We decided among these different weighting schemes through flipping simulation experiments, which we explain below.

### 3.5 Flipping simulation experiments

We design a simulation experiment where we select a percentage of the easy-to-learn samples, flip their labels, retrain the predictive models, and then use the resulting training dynamics to identify these designated mislabeled samples. These flipping simulation experiments serve two aims: to check if it is possible to identify mislabeled samples and to test different epoch weighting schemes to discover the most appropriate. In these experiments, we flipped 5% and 10% of easy-to-learn samples and repeated this four times for these two setups.

### 3.6 External evidence aggregated to support predicted misannotated lncRNAs

For candidate misannotated lncRNAs, we aggregated several sources of external data sources. Below we provide details on these data sources:

**Ribo-seq and mass spectrometry data aggregated by sORFs.org:** sORFs.org (Olexiouk *et al.*, 2018) is a database of sORFs identified by analyzing 34 human Ribo-seq datasets. It provides precomputed FLOSS, ORFscore and PhyloP scores for these sORFs. Moreover, it also incorporates publicly available mass spectrometry evidence that proves the translation of sORFs into micropeptides. The cutoffs used for FLOSS, ORFscore and PhyloP scores, the PeptideShaker score, as well as the *P*-value for Ribo-seq data used in this work are derived from this source.

**Protein homology and domain search using BLAST and hmmer:** To find homology of candidate misannotated lncRNAs to known proteins, we ran BLASTx with default parameters on the non-redundant protein sequences database (Altschul *et al.*, 1990). We used hmmer (Eddy, 2011) function hmmscan (default parameters, *E*-value for best domain < 0.05) with Pfam (Mistry *et al.*, 2021) to search for known domains in misannotated lncRNA candidates.

**Coding potential prediction using external tools:** In order to gauge the coding potential of misannotated lncRNAs, we ran 4 external coding potential prediction tools: CNIT (Guo *et al.*, 2019), CPC2 (Kang *et al.*, 2017), CPAT (Wang *et al.*, 2013) and RNAsamba (Camargo *et al.*, 2020). We used the respective webservers that allow for submission of a batch of sequences. We used default parameter settings.

**Structure prediction using AlphaFold2:** We extracted ORFs from 6 misannotated candidates using code provided by Stewart *et al.* (2017) and then ran AlphaFold2 (Jumper *et al.*, 2021) to check for any structures predicted with high confidence. For each residue within an ORF, AlphaFold2 produces a confidence score called pLDDT, which ranges between 0 and 100, with higher scores indicating higher confidence. We repeated the same process with nine randomly picked non-coding RNAs from the Easy-to-Learn region, i.e. nine RNAs for which we have high confidence that the ground truth label of non-coding is correct. We obtained a total of 146 ORFs from the 6 misannotated candidates highlighted in the paper and 42 ORFs from the 9 random true lncRNAs. We ran AlphaFold2 on all ORFs and then compared the distribution of pLDDT values for ORFs from true lncRNAs and misannotated lncRNAs.

## 4 Discussion

### 4.1 Training dynamics of deep learning models can be used to identify misannotated lncRNAs

We first evaluate the deep learning models’ predictive performance on distinguishing coding and non-coding RNA sequences. Prediction performances calculated on the held-out test set for the models trained are provided in Supplementary Table S2, Supplementary Text. The LSTM model achieves the highest classification performance with 94% AUC and 96% AUPR. The CNN model follows with 93% AUC and 95% AUPR, while the transformer achieves 91% AUC and 93% AUPR. The F1-Scores for the coding class are slightly better than or equal to F1-Scores for the non-coding class. We next employ these models to discover possibly misannotated lncRNAs in the underlying dataset.

**Fig. 3.**
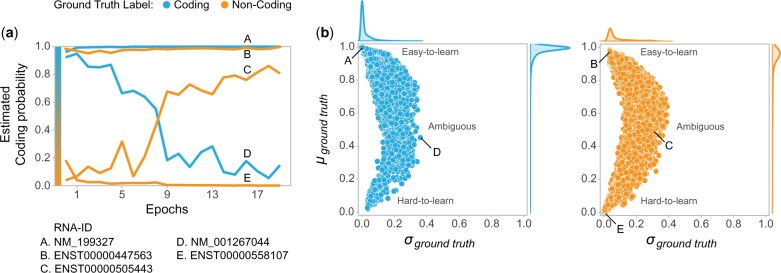
Training dynamics of deep learning models can be used to identify misannotated lncRNAs. **(a)** Estimated coding probability across all training epochs shown for five example RNAs for the LSTM model. We expect coding and non-coding RNAs to have high and low coding probabilities respectively; this is the case for examples A and E. We are interested in lncRNAs—like B (ENST00000447563)—which have consistently high estimated coding probability, despite having the ground truth-label ‘Non-coding’. C and D show examples of ambiguous samples, i.e. they show a large change in estimated coding probabilities as model training progresses, so we are not sure whether they are mislabeled or not. **(b)** Mean (*y*-axis) and standard deviation (*x*-axis) of ground truth class probability predictions across all training epochs can be used to determine mislabeled samples. The candidate misannotated RNAs are those in the hard-to-learn region i.e. RNAs with low mean and standard deviation for the ground truth class probability

Having evaluated the CNN, LSTM and Transformer models for their ability to distinguish between coding RNA and non-coding RNA, we retrain the models using all data and inspect the resulting training dynamics for each RNA. We track the coding probability predictions for each input RNA transcript at each epoch during the training. Figure 3a exemplifies the predictions for the coding probability for five different types of RNAs across all training epochs for the LSTM model. Two examples of correctly annotated coding and non-coding RNA (A, E respectively) are shown in Figure 3a, the coding probability for A and E is consistently high and low, respectively; such RNAs are very likely to be correctly annotated. Figure 3a C and D illustrate ambiguous cases, i.e. these RNAs show a large change in estimated coding probabilities as model training progresses. For this work, the most interesting cases are RNAs like B: the coding probability predictions for B (ENST00000447563)—an RNA annotated as long non-coding (ground-truth)—are consistently high. In other words, as model training progresses, this RNA is invariably classified as coding. This is an example of a putative misannotated non-coding RNA discovered by examining the training dynamics of deep learning models. It was recently shown that ENST00000447563, in fact, codes for a protein (Hartford and Lal, 2020).

Figure 3b expands upon this idea: calculating the mean and standard deviation of predicted probability for the ground-truth class across all training epochs provides a measure of identifying misannotated lncRNAs. lncRNAs in the hard-to-learn region of Figure 3b are considered candidate misannotated lncRNAs. These samples have a low mean and standard deviation for the predicted probability of the ground-truth class overall training epochs. In other words, RNAs that fall in this region are consistently classified into the non-ground-truth class. It is interesting to note that most of the putative mislabeled samples have the ground-truth label ncRNA. This could be because an RNA with ground-truth ‘coding’ is unlikely to be misannotated.

To check that using the training dynamics of the models helps identify mislabeled samples, we designed a computational experiment. We flipped the training labels for 5 and 10 percent of the easy-to-learn and ambiguous samples. After flipping the class labels, we retrain the LSTM model. Then, we assessed the model’s ability to identify the samples for which we deliberately flipped the class labels, i.e. which we know are mislabeled examples. Using these simulation experiments, we tried out different strategies for calculating the mean and standard deviation of predictions over epochs. See the Methods section for more details. Based on the percentage of mislabeled samples that were not discovered by a given scheme, we find that it is best to used all epochs and weigh them equally, see Supplementary Figure S2. Schemes that only use the last 20% of epochs had the worst performance.

**Fig. 4.**
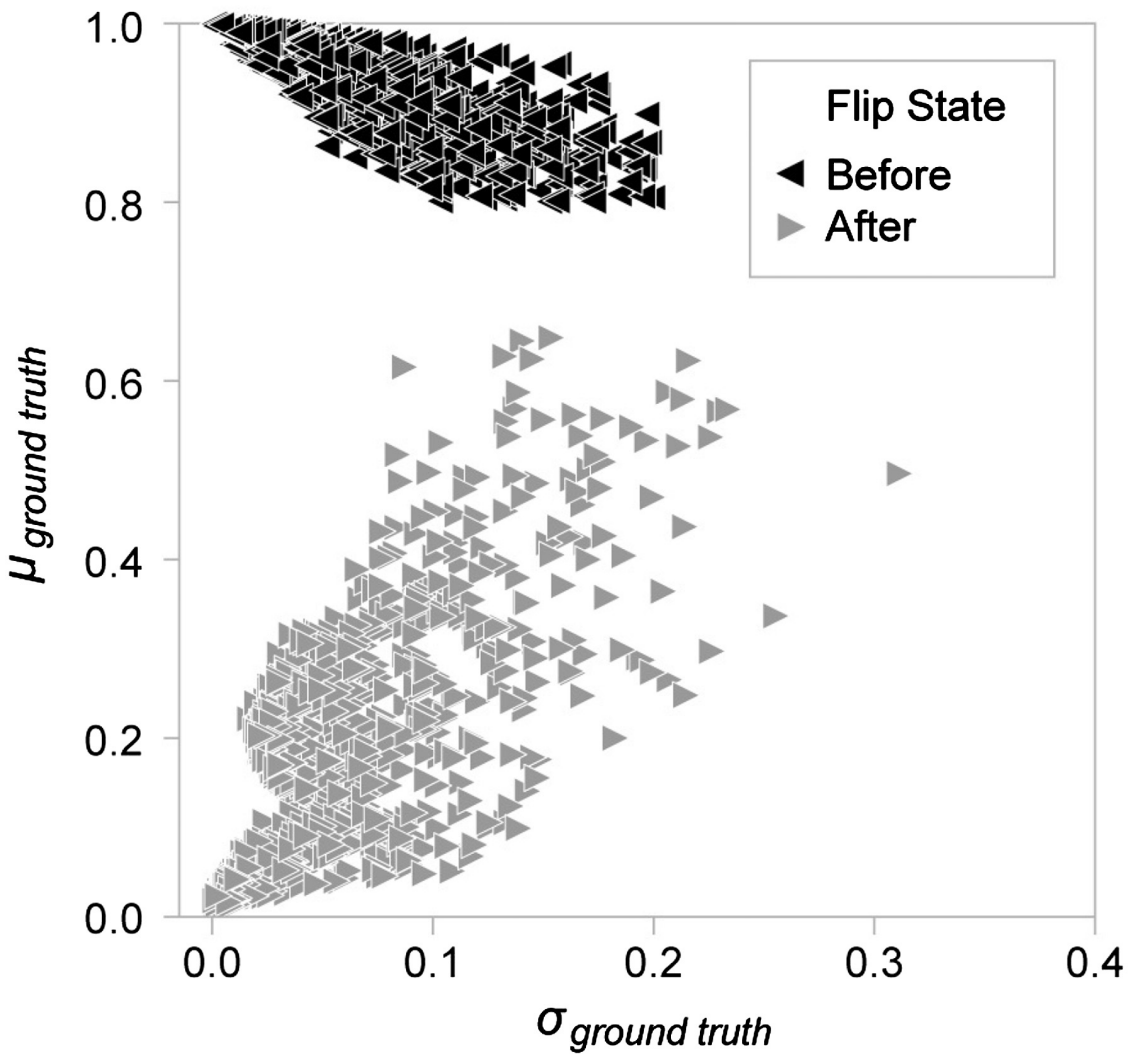
Class label flip experiments results. To illustrate that training dynamics can be used to identify mislabeled RNAs, we sampled 5% of the data with $\mu_{\mathrm{ground}\;\mathrm{truth}}\geq$ 0.8 and $\sigma_{\mathrm{ground}\;\mathrm{truth}}\leq$ 0.2. These are samples for which we have medium-high confidence that the ground truth labels are correct (before). After we flip the labels (coding RNAs become non-coding RNAs and vice-versa), the samples move into the hard-to-classify region of the training dynamics summary plot

Figure 4 shows that this method can successfully identify the flipped samples. Before flipping the labels, the *μ*_ground truth_ and *σ*_ground truth_ of the samples place the samples in the easy-to-learn part of the plot, i.e. they are classified into their ground truth classes with high confidence. When we flip the labels, these examples move into the hard-to-learn part of the graph, flagging them as candidate mislabeled examples (Fig. 4). With a cutoff of μ=0.6 and σ=0.4, we were able to identify 99%, 99%, 98% and 91% of the mislabeled samples, even when the labels for 1%, 5%, 10% and 20% of the data were flipped respectively.

### 4.2 Misannotated RNA sets discovered by different deep learning models have significant overlap

**Fig. 5.**
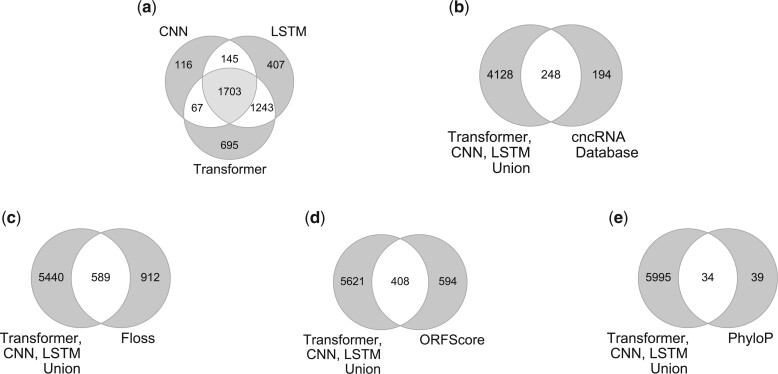
**(a)** Comparison of the set of candidate misannotated lncRNAs obtained from by CNN, LSTM and Transformer models’ training dynamics. **(b)** Comparison of misannotated lncRNAs overlapping with cncRNAdb—a manually curated resource of experimentally verified coding ncRNAs. **(c, d, e)** Comparison of misannotated lncRNAs found by CNN, LSTM and Transformer models’ training dynamics with previous Ribo-Seq data-based methods used to find misannotated lncRNAs from: **(c)** FLOSS (*P*-value ≈0), **(d)** ORFScore (*P*-value 4e–32) and **(e)** PhyloP [*P*-value (1e–32)] for the dataset from Elkon *et al.* (2015). Background set has 26 857 lncRNAs. The *P*-values represent the significance of the overlap obtained using the hypergeometric test

Figure 5a shows the overlap between the lists of misannotated ncRNAs, i.e. ncRNAs that fall in the hard-to-learn region of Figure 3a, generated by CNN, LSTM and Transformer models. Despite the difference in network architectures, the intersection of possible misannotated ncRNAs is large. The CNN model identifies the smallest number of candidate misannotated ncRNAs. It is interesting to note that the number of common candidates identified by Transformer and LSTM but not by CNN (1243 in total) is large as compared to the common candidates between CNN and Transformer only (67) and between LSTM and CNN only (145). 1703 candidates are present in the intersection of all three models and 4376 genes (6029 transcript) candidates in the union of all models (list provided in Supplementary Table S3). We proceed with the union in our downstream analysis.

### 4.3 Misannotated lncRNAs overlap significantly with manually curated, experimentally validated coding lncRNAs and with misannotated lncRNAs discovered by Ribo-Seq

To check if the candidate list of misannotated transcripts overlaps with already reported misannotated ncRNAs, we use the manually curated list of experimentally validated ncRNAs found to be coding provided by the cncRNAdb database (Huang *et al.*, 2021). We filter data to get lncRNAs found to be coding in *Homo sapiens* and compared the list to the misannotated lncRNA candidates generated in our proposed strategy. This comparison was made using Ensembl Gene IDs. Figure 5b shows the overlap between the list of misannotated lncRNAs generated by our models and the cncRNA database (Huang *et al.*, 2021). There are 248 common misannotated lncRNAs; this overlap is highly significant [hypergeometric test, *P*-value (1e–6)].

Next, we compare the overlap between our candidate misannotated lncRNAs with a high-throughput Ribo-Seq dataset using Ensembl Transcript IDs. We obtain the data on sORFs identified in the Ribo-Seq data generated by Elkon *et al.* (2015) from sORFs.org (Olexiouk *et al.*, 2018). This database provides computations of values of FLOSS (Ingolia, 2014), ORFscore (Bazzini *et al.*, 2014) and PhyloP (Miller *et al.*, 2007) metrics for RNAs identified from the Ribo-Seq data. We use RNAs annotated as lncRNAs and present in both the sequence dataset (used to train deep learning models) and the Ribo-Seq dataset in our analysis. According to previous considerations, to get the list of lncRNAs containing translated sORFs, we use the following cutoff values: ‘Good’ for the Floss-classification, ORFscore >6 and PhyloP >4 (Olexiouk *et al.*, 2018). For FLOSS, lncRNAs with a classification of ‘Good’ are considered misannotated lncRNAs; it is interesting to note that most of the lncRNAs have a ‘Good’ FLOSS score. In contrast, fewer lncRNAs are considered misannotated according to ORFScore and PhyloP.

### 4.4 Misannotated lncRNAs exist in a continuous cluster with coding RNAs

To investigate where misannotated lncRNAs lie in relation to coding and non-coding RNAs, we perform unsupervised clustering. For each transcript, we calculate features previously found useful by the community. These features include ORF length, ORF quality, nucleotide distribution, translated peptide stability, etc. (see Supplementary Table S1 in the Supplementary Text for the list of features). We use code provided by Tong and Liu (2019) for generating these features. Using these features, we apply t-distributed stochastic neighbor embedding (t-SNE) (van der Maaten and Hinton, 2008) to reveal RNA clusters.

**Fig. 6.**
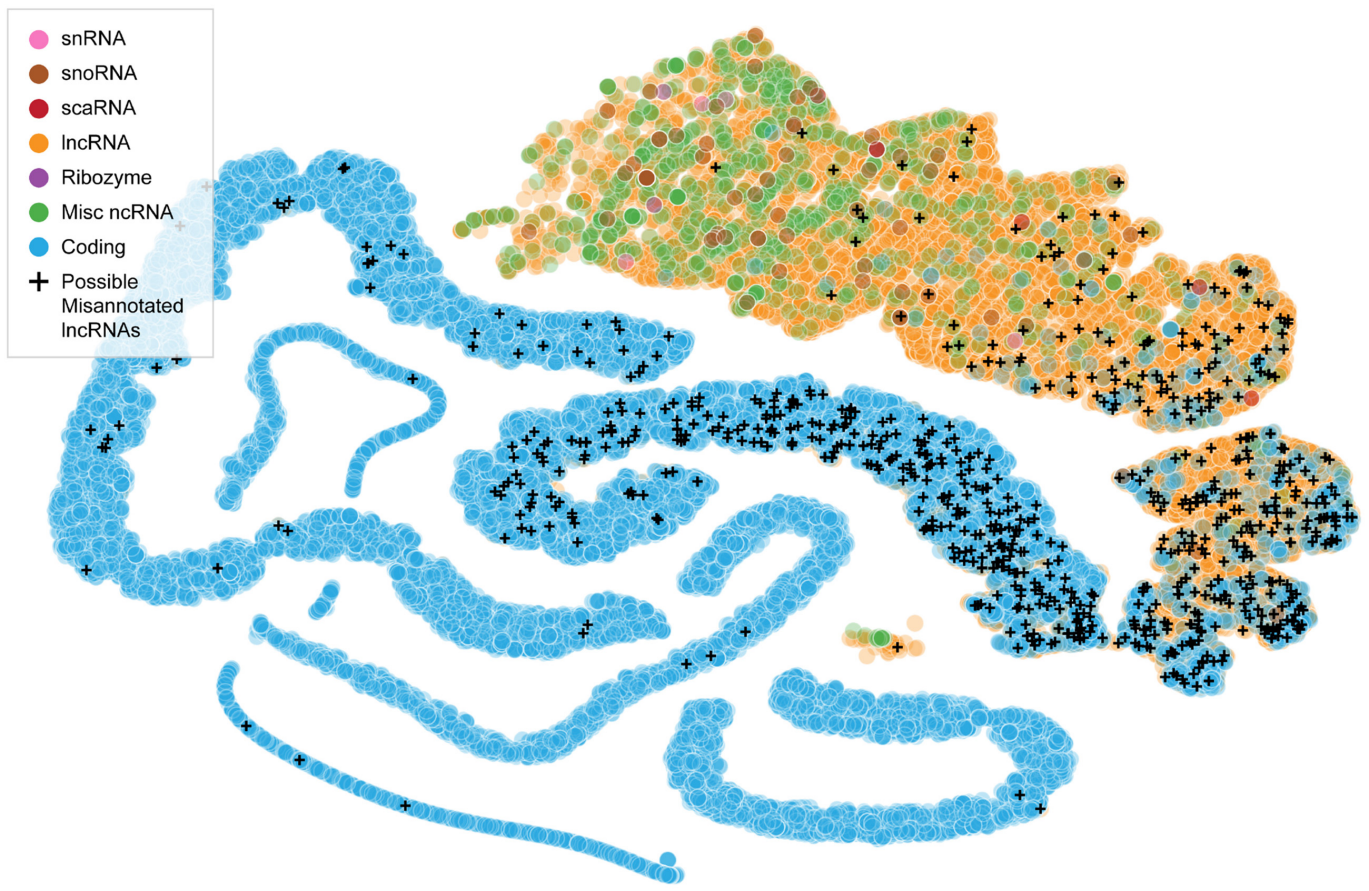
Misannotated lncRNAs exist in a continuous cluster with coding RNAs. t-SNE clusters obtained from hand-crafted features (Tong and Liu, 2019) generated from nucleotide sequences. Labels (Coding, lncRNA, etc.) are only used to visualize the clusters, not in generating the clusters. Putative misannotated lncRNAs (union of all three models) lie in a cluster contiguous with coding RNAs. There are other clusters of ncRNAs that are well separated from coding RNAs and that do not contain any putative misannotated lncRNAs

Figure 6 shows the clusters obtained by performing t-SNE on these features generated from RNA sequences. The labels of the RNAs (coding, non-coding) are not used while generating the clusters. However, based on available coding and non-coding ground-truth labels, along with the biotype information for the ncRNAs, we label each individual RNA example after the clustering. LncRNAs determined as misannotated by the different deep learning models are labeled in black; interestingly, putative misannotated lncRNAs lie in a cluster contiguous with coding RNAs. This suggests that there is indeed some continuity between coding and lncRNAs in this embedded space and that the categories might not be as mutually exclusive as we believe, which is consistent with recent research discovering that some lncRNAs encode micropeptides (Hartford and Lal, 2020). In support of this, there are clusters of non-coding RNAs (labeled Misc RNA) that are well separated from coding RNAs and that do not contain many putative misannotated lncRNAs.

### 4.5 Aggregating protein-based evidence increases confidence that misannotated lncRNAs discovered are true positives

**Fig. 7.**
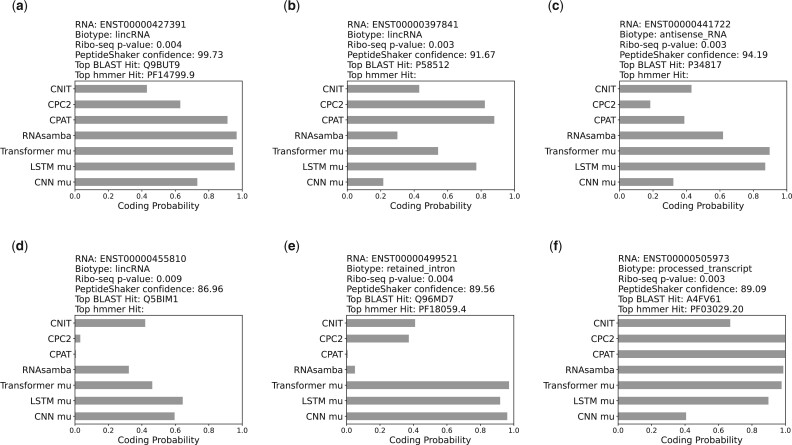
Example candidate coding lncRNAs discovered with evidence for coding potential aggregated from other sources. Ribo-Seq identifies RNAs associated with ribosomes, which are likely to be translated. Ribo-Seq *P*-values (a combined score from FLOSS, ORFScore and PhyloP) show the likelihood of the identified RNA being a true-positive. PeptideShaker analyzes publicly available MS data and provides a confidence score for each peptide. The Top BLAST Hit is the top hit from running the query in BLASTx. The Top hmmer Hit is the hmmer hit with the most significant E-value obtained by running hmmscan on the Pfam profile database. Missing values for Top hmmer Hit mean that no significant hit was found. CNIT, CPC2, CPAT and RNASamba are tools for the coding potential prediction of a given RNA

**Fig. 8.**
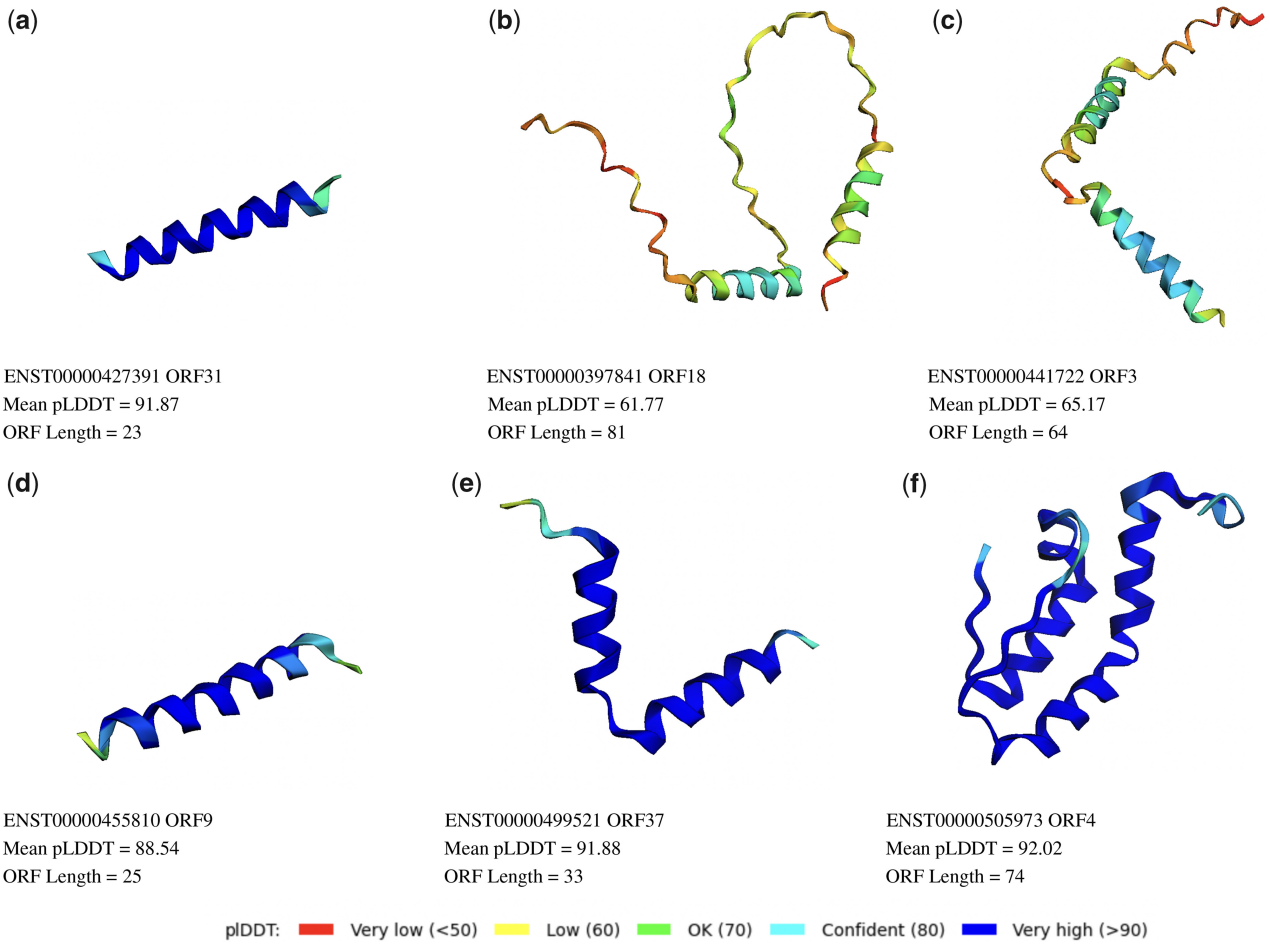
AlphaFold2 structure predictions. For misannotated RNAs shown in Figure 7, folding ORFs were extracted using code provided by Stewart *et al.* (2017) and tested for folding capability using AlphaFold2. Some ORFs with high confidence folding predictions are shown. For other folding ORFs for these RNAs, please refer to this Google Colab notebook

For the misannotated lncRNAs identified, we aggregate other useful information related to their protein coding potential. This evidence for six examples is shown in Figures 7 and 8. Figure 7 shows the transcript ID and biotype of the RNA. Confidence of being a Ribo-Seq true-positive and coding for a peptide (PeptideShaker) were obtained from Olexiouk *et al.* (2018). We ran BLASTx (default parameters, non-redundant protein sequences database) to find similarity to known proteins (Altschul *et al.*, 1990) (detailed BLAST results provided in Supplementary Table S4). The top most significant hit protein ID is shown. We ran hmmscan function from hmmer (Eddy, 2011) using Pfam (Mistry *et al.*, 2021) as the profile database to search for known domains. Furthermore, we used existing tools [CNIT (Guo *et al.*, 2019), CPC2 (Kang *et al.*, 2017), CPAT (Wang *et al.*, 2013) and RNASamba (Camargo *et al.*, 2020)] to calculate the coding potential prediction for each lncRNA. Transformer mu, LSTM mu and CNN mu refer to the average coding probability across all epochs as determined by the models trained in this work. All the examples shown in Figure 7 have high confidence scores from Ribo-Seq and MS data, as well as very significant BLAST hits. CNIT, CPC2, CPAT and RNASamba predict high coding probability for some lncRNAs. Figure 7e shows an example for which the coding probabilities predicted by CNIT, CPC2, CPAT and RNASamba are low, but for which the models trained in this work predict high coding probability across epochs.

Next, we extracted ORFs from misannotated lncRNAs shown in Figure 7 and used code provided by Jumper *et al.* (2021) to predict the folding potential for each ORF. We found many ORFs within the misannotated lncRNAs tested folded into structures with pLDDT consistently high values. To check if these values are higher than what can be expected by random, we conducted two analyses. First, we incorporated data from Monzon *et al.* (2022), who ran AlphaFold2 on randomly generated protein sequences of varying lengths. We obtained the pLDDT values for random protein sequences from this work and compared those with the mean pLDDT values for ORFs from selected lncRNAs in Figure 7. We found that the mean pLDDT values we obtained for ORFs within the missanotated lncRNAs are higher than those for random sequences (Supplementary Fig. S2). As a second analyses, we randomly picked 9 non-coding RNAs from the easy-to-learn region for which we have high confidence that the ground truth label of non-coding is correct. We extracted ORFs from these ncRNAs and obtained a total of 42 ORFs. We ran AlphaFold2 on these 42 ORFs and then compared the distribution of pLDDT values for ORFs from true lncRNAs and misannotated lncRNAs. 9% and 19% of ORFs from true and misannotated lncRNAs respectively had pLDDT values >80. These results are shown in Supplementary Figure S3. Examples of high confidence folding ORF from each lncRNA shown in Figure 7 are shown in Figure 8. An extensive list of folded structures is available in the Google Colab notebook.

Interestingly, many of the structures we find are alpha helices or combinations of alpha helices. It has earlier been reported that many small ORFs tend to display amino acid composition similar to transmembrane alpha-helices (Aspden *et al.*, 2014). Moreover, there is evidence of a small peptide that is coded by a lncRNA that binds to the groove of a membrane pump (Anderson *et al.*, 2015).

## 5 Conclusion

This article aims to detect misannotated lncRNAs using deep learning models’ training dynamics. We analyze the resulting candidate list of misannotated lncRNAs in light of various experimental evidence. The training dataset, comprising nucleotide sequences of coding and non-coding RNA, is used to train CNN, LSTM and Transformer models. Coding and non-coding prediction probabilities across epochs for every RNA sample are then used to detect the possible misannotated RNAs. LncRNAs with low mean and standard deviation for the non-coding class are designated as the candidate misannotated RNAs.

Although the architectures we used to distinguish between coding and non-coding RNAs employed are different, many possibly misannotated lncRNAs are identified by all three different deep learning methods. Moreover, when we compare the misannotations discovered here to previous methods to detect misannotated lncRNAs from a list obtained from Ribo-Seq data, we see a large overlap between two of the methods. It is also interesting to note that our method shows high overlap with a manually curated list of misannotated lncRNAs. Therefore, we suggest that this approach offers promising potential to assist experimental efforts in characterizing possible peptides encoded by lncRNAs. There are several possible directions for future work. First, comparing the misannotated lncRNAs obtained from models here with Ribo-Seq datasets from different cell types would provide interesting results on the cell-line specificity of misannotated lncRNAs. In this work, we used of FLOSS, ORFscore and PhyloP, future work can focus on using newer methods for identifying true positives from Ribo-Seq data. Second, this approach can be used to curate datasets used for training coding potential predictors. Third, we discovered several misannotated coding RNAs, which might be non-coding isoforms of coding genes. Study of these can be useful in improving annotations of coding genes as well. Finally, future work can focus on experimental validation of the misannotated lncRNAs discovered here, including whether they fall into the bifunctional or coding and non-coding RNA (cncRNA) category (Huang *et al.*, 2021).

Our work analyzed some promising candidates’ coding evidence using external coding potential tools, protein sequence similarity to existing coding genes, and potential foldability. We find candidates with high sequence similarity to protein subsequences, and our predictions using AlphaFold2 revealed high confidence folded structures. Micropeptides can stabilize protein assemblies and modify the activity of larger proteins (Anderson *et al.*, 2015; Steinberg and Koch, 2021). Using the BLAST hit results and the structure predictions, it will be interesting to investigate if these peptides can interact with the BLAST hits’ interactors we have found, which is also an interesting direction for future work.

*Financial Support*: none declared.

*Conflict of Interest*: none declared.

## Supplementary Material

btac821_Supplementary_Data

## References

[btac821-B1] Altschul S.F. et al (1990) Basic local alignment search tool. J. Mol. Biol., 215, 403–410. 2231712 10.1016/S0022-2836(05)80360-2

[btac821-B2] Anderson D.M. et al (2015) A micropeptide encoded by a putative long noncoding RNA regulates muscle performance. Cell, 160, 595–606. 25640239 10.1016/j.cell.2015.01.009 PMC4356254

[btac821-B3] Anfossi S. , Calin G.A. (2020) When non-coding is not enough. J. Exp. Med., 217. 10.1084/jem.20192009 PMC7062537 32023341

[btac821-B4] Aspden J.L. et al (2014) Extensive translation of small open reading frames revealed by Poly-Ribo-Seq. Elife, 3, e03528. 25144939 10.7554/eLife.03528 PMC4359375

[btac821-B5] Baek J. et al (2018) LncRNAnet: long non-coding RNA identification using deep learning. Bioinformatics, 34, 3889–3897. 29850775 10.1093/bioinformatics/bty418

[btac821-B6] Batista P.J. , Chang H.Y. (2013) Long noncoding RNAs: cellular address codes in development and disease. Cell, 152, 1298–1307. 23498938 10.1016/j.cell.2013.02.012 PMC3651923

[btac821-B7] Bazzini A.A. et al (2014) Identification of small ORFs in vertebrates using ribosome footprinting and evolutionary conservation. EMBO J.., 33, 981–993. 24705786 10.1002/embj.201488411 PMC4193932

[btac821-B8] Camargo A.P. et al (2020) RNAsamba: neural network-based assessment of the protein-coding potential of RNA sequences. NAR Genom. Bioinform., 2, lqz024. 33575571 10.1093/nargab/lqz024 PMC7671399

[btac821-B9] Chekulaeva M. , Rajewsky N. (2019) Roles of long noncoding RNAs and circular RNAs in translation. Cold Spring Harb. Perspect. Biol., 11, a032680. 30082465 10.1101/cshperspect.a032680 PMC6546045

[btac821-B10] Choi S.-W. et al (2019) The small peptide world in long noncoding RNAs. Brief. Bioinform., 20, 1853–1864. 30010717 10.1093/bib/bby055 PMC6917221

[btac821-B11] Couso J.-P. , Patraquim P. (2017) Classification and function of small open reading frames. Nat. Rev. Mol. Cell Biol., 18, 575–589. 28698598 10.1038/nrm.2017.58

[btac821-B12] Eddy S.R. (2011) Accelerated profile HMM searches. PLoS Comput. Biol., 7, e1002195. 22039361 10.1371/journal.pcbi.1002195 PMC3197634

[btac821-B13] Elkon R. et al (2015) Myc coordinates transcription and translation to enhance transformation and suppress invasiveness. EMBO Rep., 16, 1723–1736. 26538417 10.15252/embr.201540717 PMC4687422

[btac821-B14] Guo J.-C. et al (2019) CNIT: a fast and accurate web tool for identifying protein-coding and long non-coding transcripts based on intrinsic sequence composition. Nucleic Acids Res., 47, W516–W522. 31147700 10.1093/nar/gkz400 PMC6602462

[btac821-B15] Hartford C.C.R. , Lal A. (2020) When long noncoding becomes protein coding. Mol. Cell. Biol., 40. 10.1128/MCB.00528-19 PMC7048269 31907280

[btac821-B16] Hill S.T. et al (2018) A deep recurrent neural network discovers complex biological rules to decipher RNA protein-coding potential. Nucleic Acids Res., 46, 8105–8113. 29986088 10.1093/nar/gky567 PMC6144860

[btac821-B17] Hochreiter S. , Schmidhuber J. (1997) Long short-term memory. Neural Comput., 9, 1735–1780. 9377276 10.1162/neco.1997.9.8.1735

[btac821-B18] Huang Y. et al (2021) cncRNAdb: a manually curated resource of experimentally supported RNAs with both protein-coding and noncoding function. Nucleic Acids Res., 49, D65–D70. 33010163 10.1093/nar/gkaa791 PMC7778915

[btac821-B19] Ingolia N.T. (2014) Ribosome profiling: new views of translation, from single codons to genome scale. Nat. Rev. Genet., 15, 205–213. 24468696 10.1038/nrg3645

[btac821-B20] Ingolia N.T. et al (2009) Genome-wide analysis in vivo of translation with nucleotide resolution using ribosome profiling. Science, 324, 218–223. 19213877 10.1126/science.1168978 PMC2746483

[btac821-B21] Ingolia N.T. et al (2011) Ribosome profiling of mouse embryonic stem cells reveals the complexity and dynamics of mammalian proteomes. Cell, 147, 789–802. 22056041 10.1016/j.cell.2011.10.002 PMC3225288

[btac821-B22] Ji Z. et al (2015) Many lncRNAs, 5’UTRs, and pseudogenes are translated and some are likely to express functional proteins. Elife, 4, e08890. 26687005 10.7554/eLife.08890 PMC4739776

[btac821-B23] Jumper J. et al (2021) Highly accurate protein structure prediction with AlphaFold. Nature, 596, 583–589. 34265844 10.1038/s41586-021-03819-2 PMC8371605

[btac821-B24] Kang Y.-J. et al (2017) CPC2: a fast and accurate coding potential calculator based on sequence intrinsic features. Nucleic Acids Res., 45, W12–W16. 28521017 10.1093/nar/gkx428 PMC5793834

[btac821-B25] Kingma D.P. , Ba J. (2014) Adam: A method for stochastic optimization. In: Bengio,Y. and LeCun,Y. (eds) *3rd International Conference on Learning Representations, ICLR 2015, San Diego, CA, USA, May 7-9, 2015, Conference Track Proceedings*. http://arxiv.org/abs/1412.6980.

[btac821-B26] Kong L. et al (2007) CPC: assess the protein-coding potential of transcripts using sequence features and support vector machine. Nucleic Acids Res., 35, W345–W349. 17631615 10.1093/nar/gkm391 PMC1933232

[btac821-B27] LeCun Y. et al (1989) Backpropagation applied to handwritten zip code recognition. Neural Comput., 1, 541–551.

[btac821-B28] Li L. et al (2017) Hyperband: a novel bandit-based approach to hyperparameter optimization. J. Machine Learn. Res., 18, 6765–6816.

[btac821-B29] Lu S. et al (2019) A hidden human proteome encoded by ‘non-coding’ genes. Nucleic Acids Res., 47, 8111–8125. 31340039 10.1093/nar/gkz646 PMC6735797

[btac821-B30] Makarewich C.A. et al (2018) MOXI is a mitochondrial micropeptide that enhances fatty acid β-oxidation. Cell Rep., 23, 3701–3709. 29949755 10.1016/j.celrep.2018.05.058 PMC6066340

[btac821-B31] Matsumoto A. et al (2017) mTORC1 and muscle regeneration are regulated by the LINC00961-encoded SPAR polypeptide. Nature, 541, 228–232. 28024296 10.1038/nature21034

[btac821-B32] Miller W. et al (2007) 28-way vertebrate alignment and conservation track in the UCSC genome browser. Genome Res., 17, 1797–1808. 17984227 10.1101/gr.6761107 PMC2099589

[btac821-B33] Mistry J. et al (2021) Pfam: the protein families database in 2021. Nucleic Acids Res., 49, D412–D419. 33125078 10.1093/nar/gkaa913 PMC7779014

[btac821-B34] Monzon V. et al (2022) Folding the unfoldable: using AlphaFold to explore spurious proteins. Bioinform. Adv., 2, vbab043. 36699409 10.1093/bioadv/vbab043 PMC9710616

[btac821-B35] Nelson B.R. et al (2016) A peptide encoded by a transcript annotated as long noncoding RNA enhances SERCA activity in muscle. Science, 351, 271–275. 26816378 10.1126/science.aad4076 PMC4892890

[btac821-B36] Ng P. (2017) dna2vec: Consistent vector representations of variable-length k-mers. *arXiv preprint arXiv:1701.06279.*

[btac821-B37] Olexiouk V. et al (2018) An update on sORFs. org: a repository of small orfs identified by ribosome profiling. Nucleic Acids Res., 46, D497–D502. 29140531 10.1093/nar/gkx1130 PMC5753181

[btac821-B38] Rinn J.L. , Chang H.Y. (2012) Genome regulation by long noncoding RNAs. Annu. Rev. Biochem., 81, 145–166. 22663078 10.1146/annurev-biochem-051410-092902 PMC3858397

[btac821-B39] Ruiz-Orera J. et al (2014) Long non-coding RNAs as a source of new peptides. Elife, 3, e03523. 25233276 10.7554/eLife.03523 PMC4359382

[btac821-B40] Slavoff S.A. et al (2013) Peptidomic discovery of short open reading frame–encoded peptides in human cells. Nat. Chem. Biol., 9, 59–64. 23160002 10.1038/nchembio.1120 PMC3625679

[btac821-B41] Steinberg R. , Koch H.-G. (2021) The largely unexplored biology of small proteins in pro-and eukaryotes. FEBS J., 288, 7002–7024. 33780127 10.1111/febs.15845

[btac821-B42] Stewart Z.K. et al (2017) Transcriptomic investigation of wound healing and regeneration in the cnidarian calliactis polypus. Sci. Rep., 7, 41458–41411. 28150733 10.1038/srep41458 PMC5288695

[btac821-B43] Swayamdipta S. et al (2020) Dataset cartography: mapping and diagnosing datasets with training dynamics. In: *Proceedings of EMNLP*. https://arxiv.org/abs/2009.10795.

[btac821-B44] Tong X. , Liu S. (2019) CPPred: coding potential prediction based on the global description of RNA sequence. Nucleic Acids Res., 47, e43. 30753596 10.1093/nar/gkz087 PMC6486542

[btac821-B45] Tong X. et al (2020). CPPred-sORF: Coding potential prediction of sorf based on non-aug. *BioRxiv.*

[btac821-B46] Ulitsky I. , Bartel D.P. (2013) lincRNAs: genomics, evolution, and mechanisms. Cell, 154, 26–46. 23827673 10.1016/j.cell.2013.06.020 PMC3924787

[btac821-B47] Vaswani A. et al (2017) Attention is All You Need. In: *Proceedings of the 31st International Conference on Neural Information Processing Systems, Long Beach, California, USA*. Curran Associates Inc., Red Hook, NY, USA. pp. 6000–6010. 10.5555/3295222.3295349.

[btac821-B48] van der Maaten L. , Hinton G. (2008) Visualizing data using t-SNE. J. Machine Learn. Res., 9, 2579–2605.

[btac821-B49] Wang L. et al (2013) CPAT: coding-potential assessment tool using an alignment-free logistic regression model. Nucleic Acids Res., 41, e74. 23335781 10.1093/nar/gkt006 PMC3616698

[btac821-B50] Wang Y. et al (2020) LNCRNA-encoded polypeptide ASRPS inhibits triple-negative breast cancer angiogenesis. J. Exp. Med., 217, e20190950. 10.1084/jem.20190950 PMC7062514 31816634

[btac821-B51] Zhu M. , Gribskov M. (2019) MiPepid: micropeptide identification tool using machine learning. BMC Bioinformatics., 20, 559. 31703551 10.1186/s12859-019-3033-9 PMC6842143

