# Supplementary material for: Discovering misannotated lncRNAs using deep learning training dynamics
Source: Bioinformatics. 2022 Dec 26;39(1):btac821. doi: 10.1093/bioinformatics/btac821 (PMC9825752; doi:10.1093/bioinformatics/btac821)
Supplement: btac821_Supplementary_Data [file btac821_supplementary_data.zip › coding_lncrna_submission2022_supplem.pdf]

Supplementary Text for Discovering Misannotated lncRNAs using Deep Learning Training Dynamics

1 Prediction performance of classifying coding vs. non-coding RNAs

Table S1: The test performances of the different models trained to classify long non-coding RNAs and coding RNAs. AUC and AUPR are micro-averaged.

	AUC	AUPR		Precision	Recall	F1-Score
**LSTM**	0.94	0.96	Non-Coding	0.93	0.95	0.94
			Coding	0.95	0.94	0.94
**CNN**	0.93	0.95	Non-Coding	0.93	0.92	0.93
			Coding	0.93	0.94	0.94
**Transformer**	0.91	0.93	Non-Coding	0.93	0.88	0.90
			Coding	0.90	0.94	0.92

2 Features generated to use as input for t-SNE clustering

Table S2: The list of hand-crafted features used in t-SNE clustering.

Property	Description	Number of features
ORF length	length of the longest possible ORF	1
ORF coverage	quality of ORF	1
Fickett score	codon bias for 4 nucleotides	1
Hexamer score	hexamer usage bias	1
ORF integrity	Binary, whether ORF contains start and stop codon	1
Isoelectric point	pH at which molecule carries no net charge	1
Gravy	average hydropathicity of predicted peptide	1
Instability	estimated stability of predicted peptide	1
Composition	percentage of each of the 4 nucleotides	4
Transition	percent frequency of transition from each of nt to other nts	6
Distribution	distribution for each nt 25% intervals along sequence	20

3 Comparison of strategies for weighing epochs to identify misannotated lncRNAs

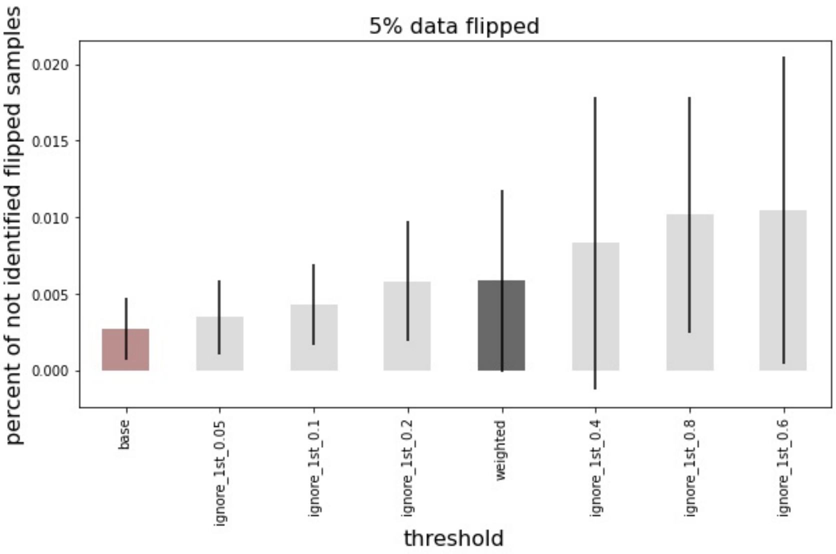

(a) 5% samples from easy-to-learn region flipped

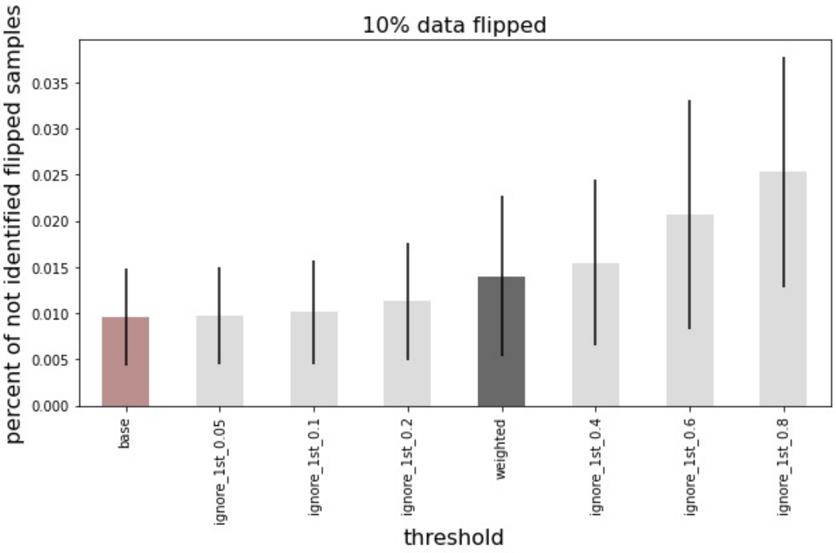

(b) 10% samples from easy-to-learn region flipped

Figure S1: **Comparison of strategies for weighing epochs to identify misannotated lncRNAs.** a) When 5% of the samples from the easy-to-learn region are flipped. b) When 10% of the samples from the easy-to-learn region are flipped. `base` refers to weighing all epochs equally; `weighted` refers to weighing epochs in proportion to epoch number such that earlier epochs get lower weight; and `ignore_1st_x` refers to ignoring first `x` = 5, 10, 20, 40, 60, or 80% epochs. The bars show the mean percent of not identified flipped samples calculated over 6 experiments, the lines shows the standard deviation calculated over 4 experiments where the randomly selected sample is changed.

4 AlphaFold Results for Noncoding Sequences

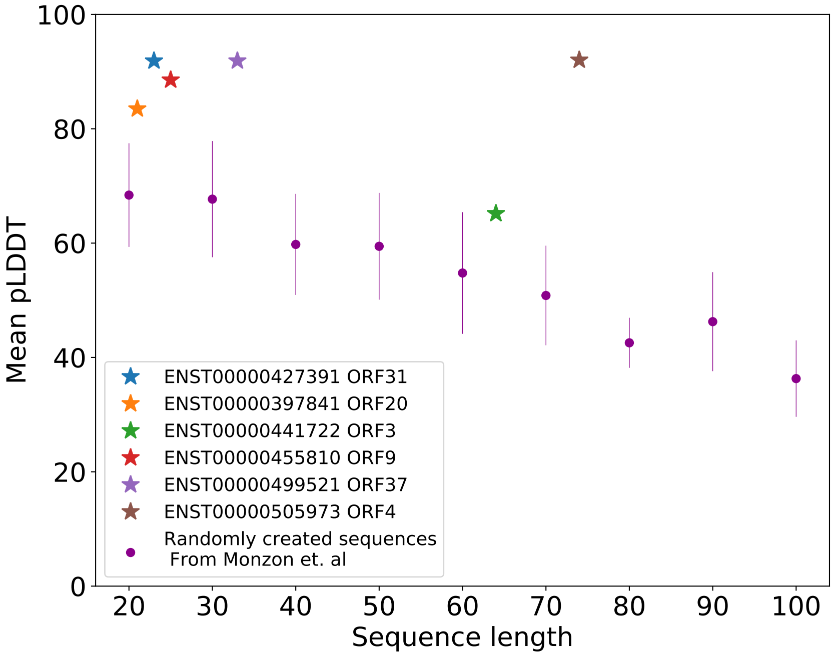

Figure S2: **ORFs within misannotated lncRNAs shown in the main text have mean pLDDT values beyond what is obtained for random sequences.** The data on mean and standard deviation of pLDDT values by sequence length for random sequences obtained from [1] study are compared with the pLDDT scores of the ORFs of the misannotated lncRNAs we obtained in this work (shown as stars).

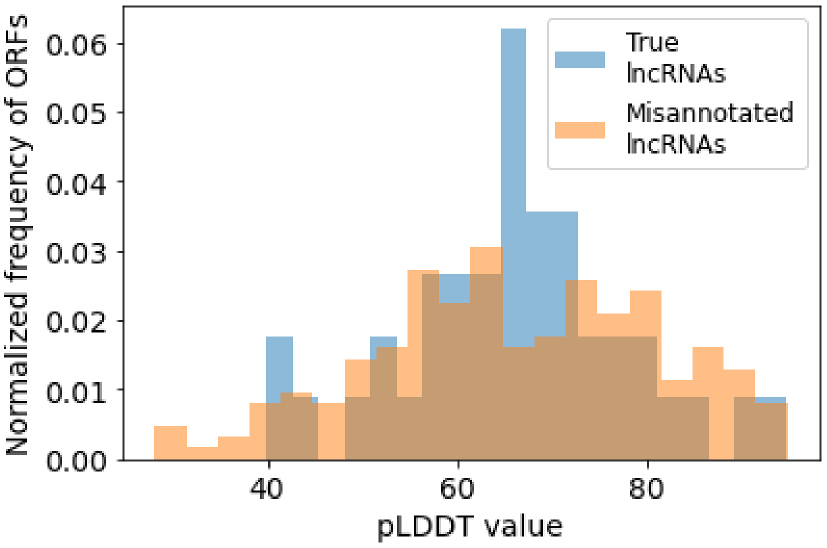

Figure S3: **Comparison of the distribution of pLDDT values of ORFs obtained from true and misannotated lncRNAs.** 9% of ORFs from true and 19% of ORFs from misannotated lncRNAs have pLDDT values > 80.

References

[1] Monzon, V., Haft, D.H., Bateman, A.: Folding the unfoldable: using AlphaFold to explore spurious proteins. Bioinformatics Advances **2**(1), vbab043 (2022)
